# Production and Characterization of Controlled Release Urea Using Biopolymer and Geopolymer as Coating Materials

**DOI:** 10.3390/polym12020400

**Published:** 2020-02-10

**Authors:** Babar Azeem, Kuzilati KuShaari, Muhammad Naqvi, Lau Kok Keong, Mohammed Khaloofah Almesfer, Zakaria Al-Qodah, Salman Raza Naqvi, Noureddine Elboughdiri

**Affiliations:** 1 CO_2_ Research Centre, Institute of Contaminant Management, Universiti Teknologi PETRONAS, Bandar Seri Iskander 32610, Malaysia; engrbabara@gmail.com; 2 Department of Chemical Engineering, Universiti Teknologi PETRONAS, Bandar Seri Iskander 32610, Malaysia; kuzilati_kushaari@utp.edu.my; 3 Department of Engineering and Chemical Sciences, Karlstad University, 65188 Karlstad, Sweden; 4 Department of Chemical Engineering, College of Engineering, King Khalid University, Abha 61411, Saudi Arabia; almesfer@kku.edu.sa; 5 Department of Chemical Engineering, Faculty of Engineering Technology, Al-Balqa Applied University, Amman 15008, Jordan; zak@bau.edu.jo or; 6 Department of Chemical Engineering, College of Engineering, Taibah University, Al-Madinah 344, Saudi Arabia; 7 School of Chemical and Materials Engineering, National University of Science and Technology, Islamabad 44000, Pakistan; salman.raza@scme.nust.edu.pk; 8 Department of Chemical Engineering, College of Engineering, University of Ha’il, Ha’il 81451, Saudi Arabia; n.elboughdiri@uoh.edu.sa; 9 Département de Génie Chimique de Procédés, Laboratoire Modélisation, Analyse, et Commande des Systèmes, Ecole Nationale d’Ingénieurs de Gabès (ENIG), Rue Omar Ibn-Elkhattab, Gabès 6011, Tunisia

**Keywords:** biopolymers, controlled-release urea, fluidized-bed coating, nitrogen release, pollution control, green fertilizers, rotary fluidized bed coater

## Abstract

Synthetic polymers-based controlled release urea (CRU) leaves non-biodegradable coating shells when applied in soil. Several alternative green materials are used to produce CRU, but most of these studies have issues pertaining to nitrogen release longevity, process viability, and the ease of application of the finished product. In this study, we utilized tapioca starch, modified by polyvinyl alcohol and citric acid, as coating material to produce controlled release coated urea granules in a rotary fluidized bed equipment. Response surface methodology is employed for studying the interactive effect of process parameters on urea release characteristics. Statistical analysis indicates that the fluidizing air temperature and spray rate are the most influential among all five process parameters studied. The optimum values of fluidizing air temperature (80 °C), spray rate (0.13 mL/s), atomizing pressure (3.98 bar), process time (110 min), and spray temperature (70 °C) were evaluated by multi-objective optimization while using genetic algorithms in MATLAB^®^. Urea coated by modified-starch was double coated by a geopolymer to enhance the controlled release characteristics that produced promising results with respect to the longevity of nitrogen release from the final product. This study provides leads for the design of a fluidized bed for the scaled-up production of CRU.

## 1. Introduction

Pristine urea is vulnerable to losses through ammonia volatilization, leaching, and surface runoff [1]. The application of controlled release urea (CRU) is an abatement strategy for avoiding this economic loss and preventing water eutrophication (via urea leaching) and hazardous nitrous emissions into the stratosphere [2]. The CRU based on synthetic polymers produced promising results in terms of longevity of nitrogen release [3,4,5]. However, there are many drawbacks with respect to cost, non-biodegradability, operational feasibility, and ease of application of the final product [6].

Recently, significant interest is triggered in this research niche for the development of CRU while using bio-based polymers as coating materials, such as starch [7], lignin [8], cellulose [9], and bio-based polyurethane [10]. These materials are not suitable for the production of CRU unless modified with some appropriate crosslinker, plasticizer, or stabilizer, due to their poor mechanical and thermophysical properties [11,12]. Despite the chemical or physical modification of these materials, most of such studies could not yield promising results in terms of either (i) effective controlled release characteristics, or (ii) overall cost of production, or (iii) viability for scaled-up production, or (iv) ease of application of the finished product [1]. For example, different starches were modified by (i) borax/urea [13], (ii) polysulfone [14], and (iii) polyvinyl alcohol/boric acid [15], and used as coating materials to produce CRU with nitrogen release time as low as 0.05, 5.0, and 8.0 h, respectively. The development of starch based films in which urea fertilizer is impregnated into a matrix of starch (with necessary additives) is commonly reported in the literature as controlled release vehicles; however, the controlled release films have the issue of ease of application in the fields [16].The dip coating or simple immersion technique is used in various studies for the production of CRU that has the drawback of aggregates formation due to poor evaporation of solvent and the subsequent development of coating heterogeneities [6].

The production and efficacy of multiple layered CRU have also been reported as an attempt to overcome the limitations of poor release characteristics. However, most of these studies used synthetic inorganic polymers for the production of double- or triple-layered CRU posing environmental concerns. For example, three layered CRU was produced with inner, middle, and outer layers made from polyethylene, poly(acrylic acid–*co*–acrylamide), and poly(butyl methacrylate), respectively [5]. Another study reported double coated urea with inner coating film of polystyrene and outer film made of polyacrylic acid-containing urea [17]. Urea formaldehyde was used as inner coating and polyacrylic acid as outer coating film to produce CRU [18]. All of these synthetic polymers are costly and some of them are non-biodegradable.

In this study, we have addressed almost all of the major issues pertaining to the production and efficacy evaluation of CRU, such as longevity of nitrogen release, environment-friendliness, cost, viability of production for up-scaling, and ease of application of the final product. A thorough literature review suggests that there is hardly any systematic study that reports on the interactive effect of fluidized bed process parameters on the production and efficacy of modified-starch based CRU. We produced CRU while using pristine urea granules as substrate and modified-starch as a biopolymer coating material in a rotary fluidized bed machine. An evaluation of the effect of interacting process parameters on controlled release characteristics and subsequent evaluation of optimized operating conditions is reported, which can be helpful for the up-scaled production of CRU in a fluidized bed. Urea coated with modified-starch is double-coated with a geopolymer to enhance the release characteristics. The efficacy of both coated products was tested in water and soil.

## 2. Materials and Methods

### 2.1. Materials

Commercially available food grade tapioca starch was purchased from a local market (CAP Kapal ABC^®^, Penang, Malaysia). It was kept in a properly sealed container in a refrigerator at −20 °C to avoid any microbial activity. Before using it for the preparation of coating solution, it was dried in an air dryer to remove any traces of moisture. It is either blended or chemically modified with a crosslinker or plasticizer, such as polyvinyl alcohol (PVOH), to overcome weak thermoplastic properties and increase flexibility and workability of starch as a coating material. The intermolecular and intramolecular hydrogen bonding between starch and PVOH introduced by the OH^−^ groups improves the film integrity [19]. For this study, Sigma Aldrich^®^ provided analytical grade PVOH in crystalline form with a degree of hydrolysis between 87.0%–89.0%, molecular weight of 13,000–23,000 g/mol, viscosity of 3.5–4.5 cP, and a pH of 4.5–6.5.

Citric Acid is used as a crosslinker for the chemical modification of native starches to enhance their properties. When used as a crosslinker, the carboxyl groups of citric acid can combine with hydroxyl groups of starch, thus reducing the hydrophilic behavior of starch and enhancing the water resistibility of the starch films [20]. Analytical grade crystalline citric acid (purity > 99.5%) was procured from Sigma Aldrich^®^ (Selangor, Malaysia) with a molecular weight of 192.12 g/mol. For the double coating of urea granules with a geopolymer, fly ash (Class F) was procured from the local power plant and analytical grade NaOH solution (10 M) was used as an activator.

### 2.2. Methods

#### 2.2.1. Synthesis of Spray Solutions

The method for the preparation of starch/polyvinyl alcohol/citric acid based spray solution (SPCSS) is adopted from Naz et al. [13]. The food grade tapioca starch was dried at 80 °C to remove any traces of moisture until the receipt of constant weight. Five grams PVOH was dissolved in 75 mL deionized water at 90 °C. The solution was heated and stirred in deionized water in a three-neck-flask for 45 min. 5 g starch was dissolved in 25 mL deionized water and then added to the PVOH solution with subsequent stirring for 30 min. Meanwhile, five grams of citric acid were dissolved in five mL deionized water separately. Citric acid solution was added into the starch-PVOH solution after cooling it to 30 °C since the addition of citric acid into the starch solution can cause hydrolysis at elevated temperature (90 °C) and high water content. The agitation was continued for another 1 h. The final SPCSS spray solution was allowed to cool at room temperature and used for further characterization and coating runs to produce controlled release single coated urea (SCU).

Fly ash (Class F) from the local power plant was used for the preparation of geopolymer solution. NaOH solution of 10 M concentration was used as an activator. The solid to liquid (S/L) ratio between fly ash and NaOH was maintained at 3:1. Three hundred grams of fly ash and 100 g of sodium hydroxide solution were mixed and diluted with 50 mL of distilled water. The mixture was then uniformly agitated at room temperature for about 5 min until a homogenous solution was achieved. The final geopolymer based spray solution (GPSS) was used for the double coating of SCU to produce double coated urea (DCU).

#### 2.2.2. Materials’ Characterization

Viscosity measurement of spray solution (SS) is important, as it can affect mean droplet diameter, droplet penetration, droplet drying time, and agglomeration during coating operations. It is a physicochemical variable that can control the growth mechanism of granules being coated [21]. In this study, viscosity of the SPCSS biopolymer was monitored by using Brookfield Viscometer CAP 2000+ with spindle 1 and speed of 300 RPM to obtain digital display reading between 10%–100% torque while following the Brookfield Viscometer’s manual. The SPCSS dispersion in water was heated in a round bottom flask on a hot plate at 80 °C. The aliquots were drawn from the flasks periodically (0, 10, 20, 30 and 60 min.) for viscosity measurement. Three measurements were taken to obtain the average value.

Thermogravimetric analysis (TGA) is used to study thermal behavior and thermal stability of the SS when it is subjected to high temperature. In this study, Perkin Elmer Simultaneous Analyzer STA 6000 accomplished TGA. At first, the sample was weighed in a crucible in the range of 10 to 12 mg. The sample was heated from 30 to 600 °C with a heating rate of 10 °C/min. The measurements were carried out under nitrogen gas atmosphere at a flow rate of 20 mL/min. The data were recorded while using Pyris Player Data Analyzer.

The differential scanning calorimetry (DSC) is useful in the determination of glass transition temperature (*T*_g_) of the materials used. A qualitative analysis of the flexibility or rigidity of materials can also be achieved by DSC. The release characteristics of coated fertilizer are dependent on the thermal properties of the materials used; hence, it is necessary for the materials to undergo DSC. In this study, DSC Q2000 (TA Instruments) was used to determine the glass transition temperature (*T*_g_) of the SPCSS. From this analysis, we can verify the glassy and rubbery nature of the prepared biopolymer. The materials were weighed and placed in aluminum DSC pans. The samples were first cooled from room temperature to −50 °C and then held at this temperature for 150 s. This was followed by a heating scan from −25 to 250 °C at a heating rate of 10 °C/min under N_2_ atmosphere. The *T*_g_ was considered as the midpoint of the change in heat capacity.

The absorption of specific radiations by using infrared spectroscopy can identify the characteristic functional groups of macromolecules in a biopolymer. This analytical technique measures the absorption of infrared radiations by the sample materials versus wavelength. In the present work, Perkin-Elmer Spectrum One Spectrometer (Selangor, Malaysia) was used to record the FTIR spectrum of SPCSS biopolymer with following details:
**Mode****Transmittance**Wavelength range400–4000 cm^−1^No. of scans200Resolution4 cm^−1^

The organic molecules convert the infrared radiation to its vibrational and rotational energy at the aforementioned wavelength range. The absorption wavelength is a function of the bonds’ force constant and atoms’ geometry and relative mass.

The X-Ray Diffraction (XRD) analysis is generally carried out to know the crystallinity of the materials. In some cases, the effect of a crosslinker on the structure of the material has also been investigated by XRD. In this study, the XRD of SPCSS was accomplished after a complete dewatering in an air dryer at a temperature of 45 °C for 48 h until a constant weight was received for five consecutive times. Bruker D8 Advance diffractometer was used for the XRD analysis of dried, ground, and powdered samples (particle size = 100 µm). The sample grinding was done while using RestchZM 200 and sieving was carried out using Fisherbrand analytical sieves. The diffractometer consists of an auto sampler (90-position) and a LynxEye sensitive detector (linear position). The diffraction patterns were collected at a scan range of 2θ = 5°–60°, step size of 0.02°, and step time of 1.0 s.

#### 2.2.3. Production of Controlled Release Urea

**Figure 1:**
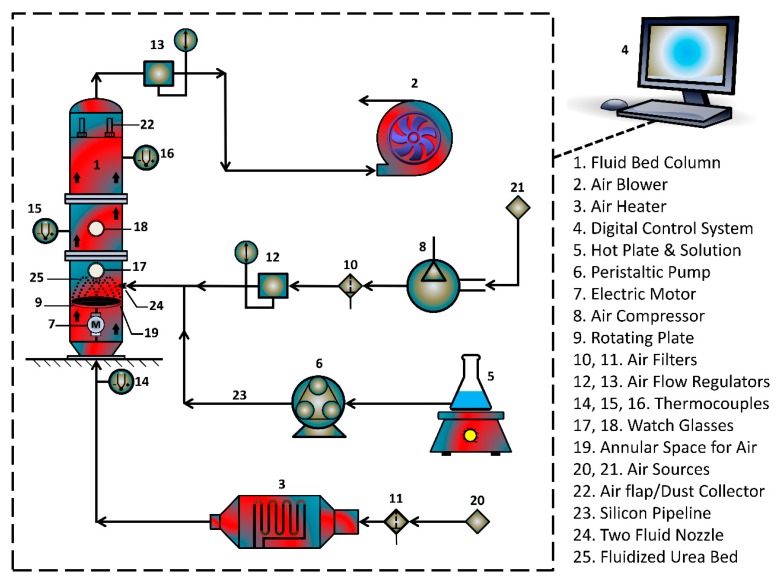
Schematic arrangement of urea coating equipment.

Single coated urea (SCU) was produced in FLP 1.5 rotary fluidized bed coater (RFBC) equipment that was provided by Wild Horse^®^, China (Jiangsu). Figure 1 shows the schematic arrangement of the equipment. Table 1 lists the characteristics of the RFBC. The RFBC is equipped with a powerful suction blower that drives the laboratory air through heater and main column of the RFBC machine, such that the substrate granules are fluidized with hot air when it passes through the annular space between machine walls and the rotating disk. The centrifugal force of rotating disk pushes the granules towards the annular space. The hot fluidizing air escapes from vent provided at the top of the main column. A peristaltic pump transports the SPCSS solution into a two fluid nozzle for the spray coating of urea granules. The temperature of different sections of the fluidized bed was noted by installing thermocouples at different heights. The control of process parameters was monitored by a digital control system. The coating of substrate granules was accomplished intermittently to avoid the unwanted agglomerates formation or defluidization due to higher viscosity of starch-based solution. For the drying of coated product, the fluidizing air flow was carried on for 15 min after every coating session while keeping the spray input off. The coated granules were subjected to curing process at 80 °C for 24 h. A total number of 50 coating runs were carried out based on the experimental design.

DCU was prepared for better release characteristics. For this purpose, SCU granules were subjected to spray coating by mechanical means while using GPSS as a coating material. GPSS was stirred uniformly at room temperature by mechanical mixing for about 5 min. until the solution was homogenous. The spray gun was assembled and then connected to the inlet air. GPSS slurry was immediately poured into the spray bowl and sprayed on the pre-weighed SCU granules for three times. The sprayed urea granules were air dried at 50 °C for 7 min. The spray coating and drying steps were repeated for 15 times to get the uniformly coated urea granules. Controlled release double coated urea (DCU) was immediately cured in a humidifier at 80 °C and 95% RH for two days. The curing in a humidifier was accomplished, because the presence of water helps in the reaction to form geopolymer networks. After two days, DCU was taken out and used for further characterization.

#### 2.2.4. Nitrogen Release Analysis

The nitrogen release characteristics (cumulative release time/release rate) were determined by the immersion of 2.0±0.01 g of each sample in 200 mL distilled water in a glass beaker and subsequent measurement of absorption in a Jasco V-630 UV-Vis Spectrophotometer after regular intervals. For every absorption measurement, 3.5 mL of the gently stirred aliquot was taken out and the total volume was maintained by the addition of the same amount of distilled water into the beaker. Every reading was taken three times and the standard curve method was used to determine total release time and subsequent release rate with respect to time. The coated products were subjected to soil burial tests to evaluate the nitrogen release characteristics in soil. For this purpose, the samples (2.0 g each) were buried 5 cm under 30% moistened soil after properly packed in a small woven mesh bag [22]. The weight loss of samples was noted after regular intervals by taking out the buried bags and measuring the weight after drying. It was continued until the receipt of a constant weight for continuous three times.

#### 2.2.5. Kinetics of Nitrogen Release

The study of release kinetics is critical to design novel controlled release fertilizers and choose appropriate encapsulating agents for tuning the release characteristics. The release of active nutrient from the core fertilizer, especially in case of a swelling membrane material, is a complex process. The transport of nutrient molecules through the coating membrane is governed by a non-constant factor, diffusion coefficient [23], which is characterized as a kinetic parameter. The diffusion coefficient of nitrogen release from SCU in this study was estimated by the following mathematical model that is derived from Fick’s second law of diffusion.

(1) $$D=\frac{l}{C_{s}\left(t-t^{\prime }\right)}\left\{\left(\frac{1}{3}rg_{t}\rho_{s}\right)+\frac{l}{6}\right\}$$

where:

*D* = diffusion coefficient (cm^2^/min.); *l* = coating thickness (cm);
*C_s_* = saturation concentration of urea (g/cm^3^); *t* = release time (min);
*t*^′^ = lag period (min.); *r* = diffusion radius (cm); and,
*g_t_* = release rate (g/min); *ρ_s_* = density (g/cm^3^).

Coating thickness (*l*) was found by using Field Emission Scanning Electron Microscope (FESEM). The radius of the swollen granule (*r*) was calculated by multiplying the radius of coated granule by 1.2. This was based on our preliminary study [24], which revealed that the degree of swelling (in terms of thickness) of the PVOH films was approximately 20%. The *t*^′^ was ignored while calculating the diffusion coefficient due to a very short lag period.

#### 2.2.6. Experimental Design and Statistical Analysis

Response Surface Methodology (RSM) is employed to study the interactive effect of different process parameters on the response variables, investigate the most and the least influential parameters, and perform the process optimization. The RSM can provide adequate and reliable measurements of the responses by the development of a mathematical model having the best fit to the data obtained. For this purpose, Central Composite Rotatable Design (CCRD) was chosen for the experimental design of five process variables while using Design Expert^®^ 8.0. The selected process variables include atomizing air pressure, fluidizing gas temperature, spray rate, spray temperature, and coating time. Before using the CCRD technique, several trial runs were performed to investigate the minimum and maximum values of the process variables, as given in Table 2. In engineering practices, it is frequently desired to minimize the cost and maximize reliability and performance. In such conflicting cases, an optimal solution for one response objective (minimizing the cost for example) might not be suitable for the other response objective (maximizing the production and reliability for example). In such a scenario, a set of optimal solutions solves the problems that can be accomplished by multi-objective optimization while using genetic algorithms. We used genetic algorithms in MATLAB^®^ to evaluate optimum process conditions by multi-objective optimization.

#### 2.2.7. Empirical Modelling of Nitrogen Release Kinetics

In this study, the curve fitting technique was used for the development of empirical correlation. Power function was used to model diffusion coefficient versus time for 100% release of nitrogen from SCU. For this purpose, the release time and corresponding diffusion coefficients of SCU samples were plotted. The curve fitting using power function generated an empirical equation of the following form:

(2) $$y=m_{0}x^{m_{1}}$$

where, *m*_0_ and *m*_1_ are the numerical constants. This equation is used to predict time for 100% release of nitrogen (*y*) while using experimental values of diffusion coefficients (*x*). The experimental and predicted values of *y* are compared and correlation coefficient and RMSE are reported as indicators of the goodness of data fitting.

## 3. Results and Discussion

### 3.1. Characteristics of Spray Solution (SPCSS)

#### 3.1.1. Viscosity

**Figure 2:**
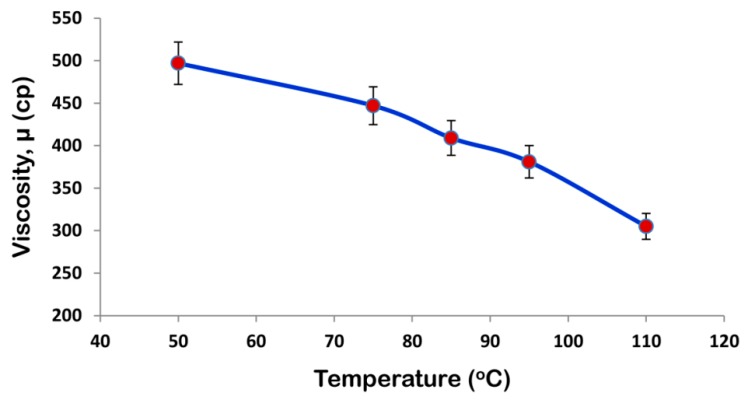
Viscosity-temperature profile for starch/polyvinyl alcohol/citric acid based spray solution (SPCSS) formulation.

The viscosity of the coating formulation plays a significant role during the coating process. The effect of temperature on viscosity of the coating formulation is also reported since coating runs are carried out at different temperatures. Figure 2 shows that viscosity of SPCSS decreases with temperature. The viscosity of SPCSS determines the droplet size during the spray process [25]. A higher viscosity at lower temperature (see Figure 2) results in the generation of relatively bigger spray droplets and fine spray droplets are produced when the solution viscosity reduces at an augmented temperature [26]. The quality of coating film highly depends on the size of spray droplets. In an ideal case, the spray droplets wet the surface of the granules, coalesce with other droplets, spread on the surface, undergo drying, and then form a uniform solid film [27]. However, the droplet size that depends mainly on the solution viscosity determines the fate of the final coating film. For instance, when the viscosity of the solution is high at lower temperature, the motion of the droplets impinged on the granule surface is sluggish due to the low velocity and less Reynolds number. A lower Reynolds number results in the poor spreading of the coating film on the surface of the granules [28]. As a result, the coating solution tends to accumulate on part of the surface, giving rise to preferential coating and coating heterogeneities. The higher the degree of coating heterogeneities on the surface of granules, the less desirable will be the controlled release characteristics [29].

In addition, the weak spreading and local accumulation of the coating material result in lower drying efficiency of the fluidizing gas increasing the vulnerability of substrate granules to undergo agglomeration phenomenon [30]. A higher degree of agglomeration can result incomplete defluidization and, hence, the collapse of the fluidized bed. The formation of temporary agglomerates results in the production of rough and porous coating films that are responsible for the poor controlled release and morphological properties of the final coated product [31]. The viscosity is lower at an elevated temperature, as shown in Figure 2. With a reduced viscosity, the spray nozzle generates fine spray droplets that facilitate proper spreading and quick drying on the surface of granules to form a uniform coating film. In this case, there are fewer possibilities for the substrate granules to undergo the agglomeration process. The addition of PVOH gives rise to the viscosity even more since starch itself is viscous. The role of temperature becomes more important in this case.

It was observed in the actual coating experiments that the agglomeration tendency rises at lower temperature, which is in accordance to the results that were presented by Keningley et al. [32] and Schaafsma et al. [33]. At a reduced temperature, the major contribution towards the initiation of agglomeration is believed to be from PVOH. With the evaporation of solvent, PVOH becomes sticky, and that is the reason why PVOH is used in many commercial applications as glue with water as a solvent [19]. PVOH has good adhesion properties, but this quality may have adverse effects during the coating process in terms of agglomeration of the granules. The agglomeration phenomenon results in rough and porous coating film, which does not only reduce the release time of the nutrient, but also results in poor mechanical properties of the final SCU.

Viscosity of the spray solution is an important parameter in urea coating. A higher viscosity reduces the impact velocity and consequent Reynolds number and Weber number of the coating film on the surface of granules. A low Reynolds number results in poor spreading of the spray droplets, giving rise to coating heterogeneities that are highly undesired. Therefore, the evaluation of an appropriate temperature is needed for the receipt of an appropriate viscosity of the spray solution. The temperature-viscosity profile that is shown in Figure 2 is helpful in deciding the appropriate temperatures for fluidizing gas and the coating solution.

#### 3.1.2. Fourier Transform Infrared Spectroscopy

**Figure 3:**
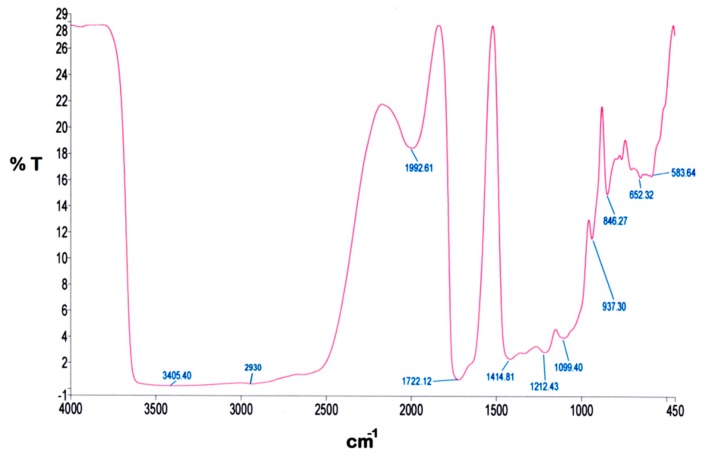
FTIR spectrum of SPCSS formulation.

Figure 3 presents the FTIR spectrum of SPCSS. The spectrum can be classified into O–H stretch bands (3000–3600 cm^−1^), C–H stretch bands (2800–3000 cm^−1^), fingerprint region (800–1500 cm^−1^), and area below 800 cm^−1^. These regions in the spectrum are characterized as starch regions [34]. The –OH stretching vibration is visible at broad absorption peak (3405 cm^−1^) [35]. In the literature, a similar broad peak is reported at 3411 cm^−1^ for the spectrum of starch modified by polyvinyl alcohol. It was reported that the width and shape of this broad peak was similar to the starch-alone spectrum [36], which indicates that the structure of hydrogen bonding in the starch-PVOH blend remains similar to what was reported for starch-alone.

The peak at 2930 cm^−1^ is associated to the C–H stretching absorption. A similar kind of peak is also reported by Lum et al. [15] at 2927 cm^−1^ and Rychter et al. [34] at 2800–3000 cm^−1^. Yin et al. [36] attributed the peak at 2931 cm^−1^ as a weak and mid-strength flexible vibration peak of –CH_2_. Since it is unlikely that all the carboxyl groups are esterified, the broad peak at 1723.91 cm^−1^ can be attributed to the C=O stretching vibration [37]. Shi et al. [37] reported that the peak at 1729 cm^−1^ might possibly be a coalescence peak that appeared due to ester bond and carboxyl group in citric acid, which is used as a crosslinker. It was further reported that this peak did not appear in the IR spectrum of starch-alone. In addition, the IR spectrum of citric acid with starch-alone and PVOH-alone also witnessed the peak at 1729 cm^−1^, which indicates that the esterification exhibits not only between citric acid and starch, but also between citric acid and PVOH. However, the esterification between citric acid and starch is said to take place more easily as compared to citric acid and PVOH [37]. The peak at 1411.20 cm^−1^ represents the C–C stretching vibration. This kind of peak at 1419 cm^−1^ is reported by Lum et al. [15]. The peaks at 1329 cm^−1^ and 845 cm^−1^ are attributed to the characteristic absorption of starch [35]. The peak at 1213 cm^−1^ appears due to the deforming vibration of –CH_2_ in –CH_2_OH [36]. Yin et al. reported a similar peak at 1236 cm^−1^. The stretching vibration C–O in C–O–H witnesses the peak at 1084 cm^−1^ [35].

The FTIR analysis of SPCSS vindicates the existence of a proper crosslinking between PVOH and citric acid (Figure 3). A substantial degree of crosslinking will offer promising resistance for the nutrient molecules to escape through the membrane grids when the coated granules are subjected to dissolution testing [15].

#### 3.1.3. Thermogravimetric Analysis

In thermal analysis, the properties of a substance are measured as a function of temperature. Thermogravimetric analysis (TGA) reports weight loss due to heating. The weight loss of a material due to heating is associated with the degradation of the material itself. Thermal analysis is important to ensure that thermal decomposition will not take place when the coating material is subjected to high temperature during the coating process and burial under the soil.

**Figure 4:**
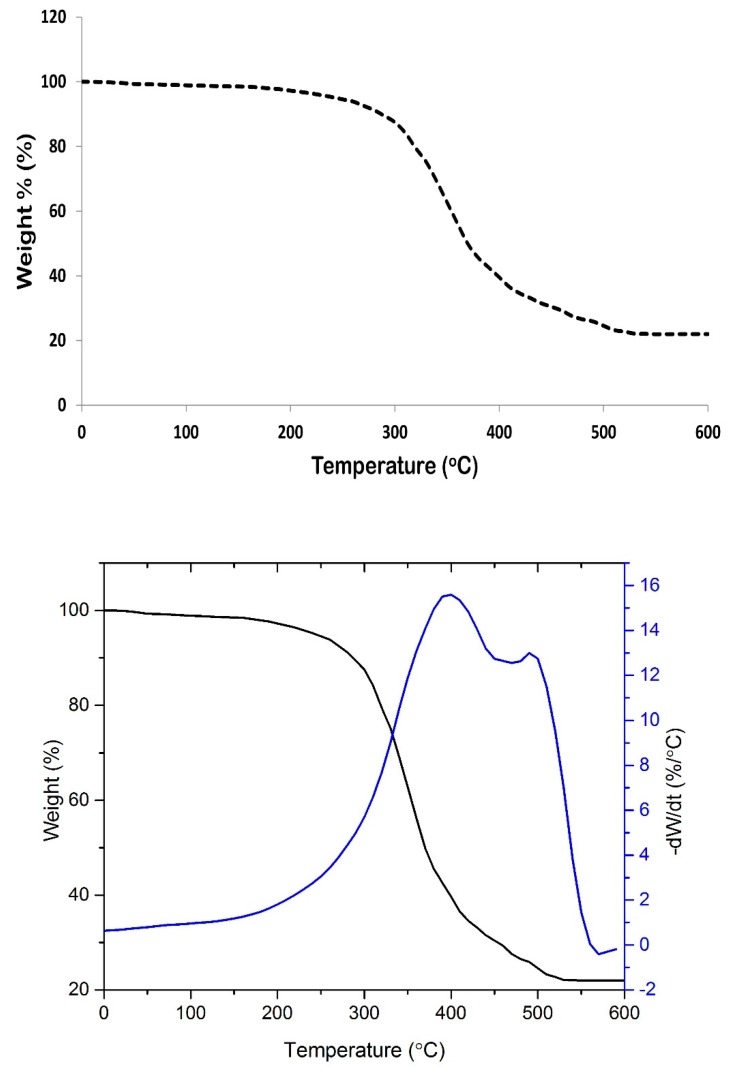
Thermogravimetric analysis/DTG (TGA/DTG) thermogram of SPCSS formulation.

Figure 4 represents the TGA thermogram of SPCSS coating formulation. The material decomposition is witnessed in two stages. The onset of decomposition is observed at around 240 °C and the complete decomposition results in a residue of 21.5% at 600 °C. The initial low magnitude—but gradual—loss of weight can be attributed to the loosely bound water molecules along with the production of volatile disintegrated matter. The major weight loss event at about 300 °C can be associated to the heat decomposition of molecules that produced small molecular carbon and hydrogen. Shi et al. [37] reported TGA analysis of PVOH-modified corn starch in the presence of citric acid as a crosslinker. The PVOH to starch ratio used was 3:1 with the addition of a 20 weight% citric acid. Shi et al. found the major weight loss event at around 280 °C and approximately 17% residue was left at 600 °C. A comparison of their work with our study shows that the thermal stability of SPCSS material used is relatively better. This might be attributed to the higher degree of crosslinking in the presence of citric acid.

In this study, the quantities of starch, PVOH, and citric acid were all equal, which might result in the formation of stronger hydrogen bonds between citric acid and starch/PVOH. Hence, thermal stability was increased. Shi et al. [37] also reported that the thermal stability of PVOH-modified starch material (with citric acid as crosslinker) is higher than PVOH-modified starch material with glycerol as the crosslinker. The weight residue of the former was also higher than the later. This was attributed to the partial crosslinking which improved the thermal stability and strong hydrogen bonding between citric acid and starch/PVOH. Consequently, the initial decomposition temperature was enhanced.

Ariyanti et al. [38] reported thermal degradation of tapioca starch at 300 °C and attributed this to complex thermal degradation of amylose and amylopectin, which is similar to the findings of the current study. However, this is not a perfect analogy, because our study uses PVOH-modified starch in the presence of citric acid as a crosslinker and not just simple starch.

Thermal degradation analysis serves two purposes. One, it helps to decide an appropriate temperature condition for fluidizing gas temperature and spray temperature. If thermal degradation temperature is unknown, coating material might be deteriorated during the coating process, which might adversely affect the coating uniformity and nitrogen release properties. Two, the information on thermal degradation temperature is also required to design CRF for their use in soil where the weather is very hot. Figure 4 indicates that the thermal degradation temperature is high enough to withstand deterioration during the coating process and hot weather conditions.

#### 3.1.4. Differential Scanning Calorimetry (DSC)

DSC analysis is another technique for examining the thermal behavior of coating formulation when it is subjected to heating and cooling runs over a range of temperature. The glass transition temperature of the material can also be determined by this technique [37]. Citric acid can be used as a crosslinker and plasticizer for the chemical modification of starches. When used as a crosslinker, the carboxyl groups of citric acid can combine with hydroxyl groups of starch, thus reducing the hydrophilic behavior of starch and enhancing the water resistibility of the starch films. The strong hydrogen bonding between the carboxyl and hydroxyl groups of citric acid and starch enhance the mechanical properties of starch films [39].

**Figure 5:**
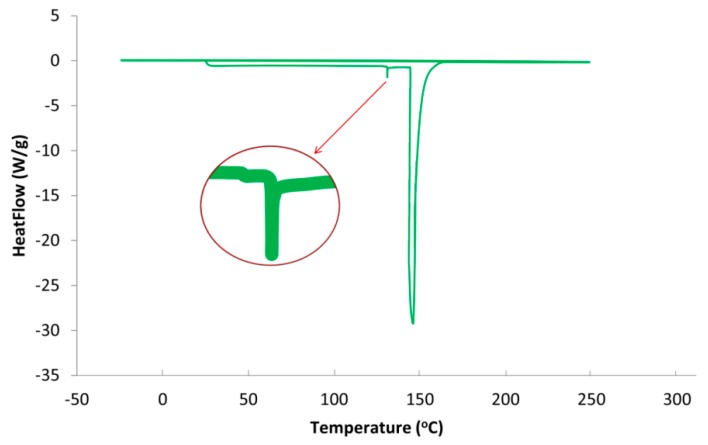
DSC thermogram of SPCSS formulation.

Figure 5 presents the DSC thermogram of SPCSS formulation. A minor endothermic peak at ~130 °C and a high endothermic peak at 148 °C is observed, while no exothermic peak appears, as shown in Figure 5. The minor peak at 130 °C can be attributed as glass transition peak (*T*_g_ = 130 °C) and the high endothermic peak at 148 °C as the melting peak (*T*_m_ = 148 °C). These results can be related to thermal behavior of starch based materials reported in the literature. It can be seen in Figure 5 that the peak for glass transition temperature (*T*_g_) is not so prominent and a zoomed-in snapshot is separately presented. It has been reported in the literature that it is not always possible for the *T*_g_ peak of starch to be visible on the DSC thermogram [37]. The non-prominence or occasional complete disappearance of the *T*_g_ peak for starch can be attributed to the engulfment of amorphous chains by the crystalline domain, the presence of H_2_O in the form of moisture, the impediment of mobility of amorphous chain segments, and the presence of inter-crystalline phases with abnormal thermal behavior [40].

The addition of citric acid positively affected the glass transition temperature. For instance, Shi et al. [37] reported a *T*_g_ value of 11.0 °C for PVOH-modified starch formulation when glycerol was used as a plasticizer. The *T*_g_ rose to 54.33 °C in the absence of glycerol and with the addition of citric acid as a crosslinker between PVOH and starch. The increase in *T*_g_ by the addition of citric acid as a crosslinker is attributed to (i) limited mobility of molecular segments due to partial crosslinking and (ii) the resistance offered by strong hydrogen bonding between citric acid and PVOH and citric acid and starch to resist the movement of the molecules. In the current study, the concentration of citric acid used as a crosslinker is higher than the one utilized by Shi et al. [37]. Therefore, the increase in *T*_g_ in current study can be attributed to the higher degree of crosslinking between citric acid and PVOH/starch that offers a higher impediment for the movement of molecules and, hence, there is a rise in *T*_g_. However, this is only true if the minor peak (zoomed-in) is considered to be a peak for the state of glass transition. If this peak is the result of some system noise, the *T*_g_ peak would be considered to be absent, as reported by various authors [40]. Some other studies have also reported a melting temperature (*T*_m_) of 150 °C for starch/modified-starch, which is close to the *T*_m_ reported in the current study [41].

DSC thermal analysis is performed to evaluate the plasticization effect of citric acid in starch and PVOH. The plasticizers reduce the glass transition temperature of the polymer matrix and prevent the formation of cracks and pin holes [42]. The addition of citric acid in the recipe decreases the glass transition temperature, which is in synchrony with the results reported in literature.

#### 3.1.5. X-Ray Diffraction (XRD)

**Figure 6:**
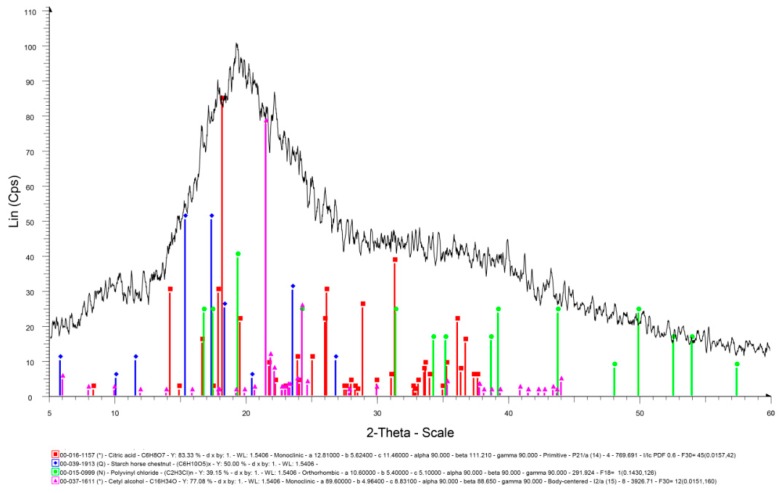
XRD spectrum of SPCSS formulation.

The X-Ray Diffraction (XRD) analysis is generally carried out to know the crystallinity of the materials [43]. In some cases, the effect of a crosslinker on structure of the material has also been investigated by XRD [44]. In this study, XRD of the coating material was accomplished after completely dewatering it. Figure 6 presents the XRD spectrum of SPCSS formulation with peaks’ assignment.

The peaks at 15.2°, 17.2°, 18.2°, 23.3°, and 26.7° of 2θ in Figure 6 represent the starch peaks. Shi et al. [37] studied the XRD spectrum of corn starch and reported similar peaks at 15°, 17°, 18°, 23°, and 26.5° of 2θ. The most intense peaks for starch are observed at 15.2° and 18.2° when compared to only a single intense peak for starch at 18°, as reported by Shi et al. [45]. The broad diffraction peak at 19.3° corresponds to PVOH. This is in synchrony with literature [45], where the broad peak at 19.3° is attributed to PVOH. The peak at 19.3° is due to the crystallinity of PVOH. The crosslinking effect of citric acid reduces the crystallinity of PVOH and, therefore, it is reported that the addition of citric acid reduced the intensity of the diffraction peak at 19.3° [37]. The peak at 31.3° appeared to be due to residual citric acid in the blend, which could not take part in the crosslinking. A similar peak is reported in the literature for residual citric acid at 31.5°.

### 3.2. Interactive Effect of Process Parameters on Urea Release Time

**Figure 7:**
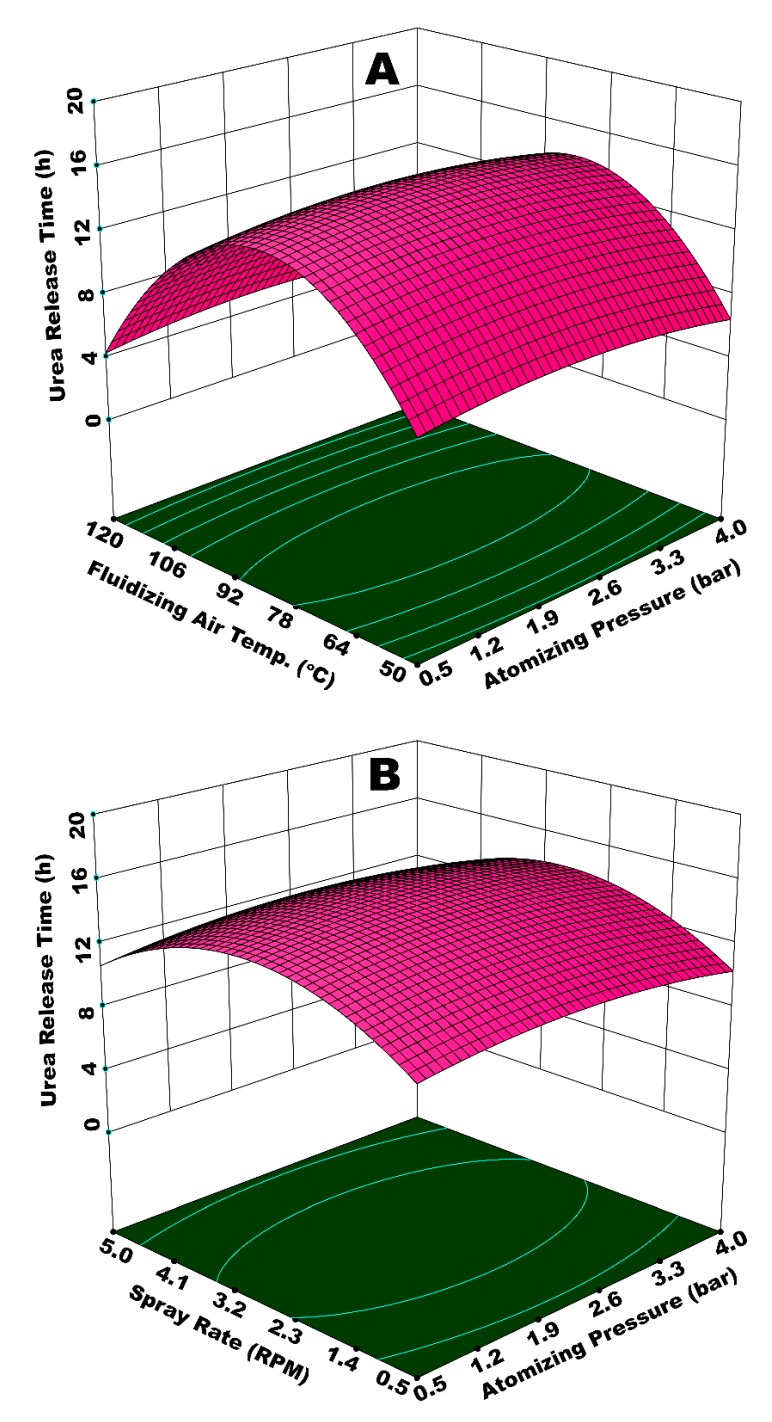
Response surfaces for (**A**) *P*_atom_ and *T*_fluid_ and (**B**) *P*_atom_ and *W*_s_ *vs* release time of urea from single coated urea (SCU).

Figure 7A illustrates the interactive effect of atomizing pressure (*P*_atom_) and fluidizing air temperature (*T*_fluid_). The response surface portrays that time for urea release gradually increases with increase in both *P*_atom_ and *T*_fluid_, reaches to a maximum, and then declines with further increase in *P*_atom_ and *T*_fluid_. It is important to understand how these process parameters individually affect the hydrodynamics of spray droplets in order to understand this behavior resulting from integrated effect of *P*_atom_ and *T*_fluid_. With increase in *P*_atom_, the diameter of spray droplets is reduced to form very fine mist [16]. Fine droplets form uniform and fine coating layer on substrate particles because of very low spray losses on the walls [16]. A uniform coating film increases the resistance of polymer film against the osmotic pressure of molten nutrient, consequently increasing the release time. However, this is true for an increase in *P*_atom_ only up to a certain level, beyond which the effect of *T*_fluid_ becomes more critical.

**Figure 8:**
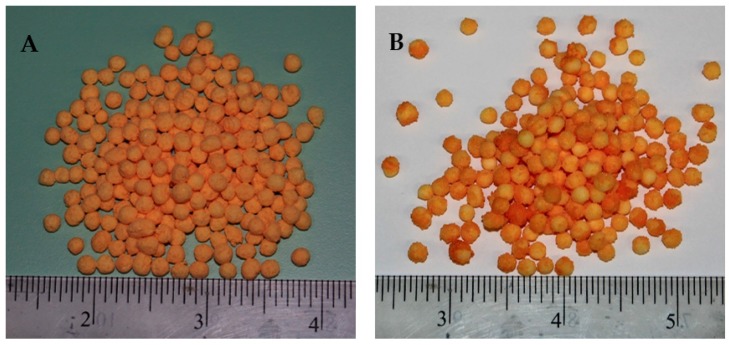
SCU samples achieved at (**A**) moderate *T*_fluid_ and *P*_atom_ and (**B**) higher *T*_fluid_ and *P*_atom_.

*T*_fluid_ significantly influences the droplet hydrodynamics. On the surface of substrate, the impingement and coalescence of fine droplets, droplets spreading, film development, solvent evaporation, and the subsequent formation of a uniform solid coating layer depends on *T*_fluid_. Initially, an increase in *T*_fluid_ positively supports the aforementioned operations, such that viscosity is reduced, which facilitates the droplet spreading and film formation. In addition, the solvent evaporation takes place quickly, which inhibits the agglomerate formation. Consequently, a uniform film engulfs the substrate, which results in an extended release time of nutrient when the SCU granules are subjected to dissolution. However, as the *T*_fluid_ exceeds 85 °C and *P*_atom_ increases beyond 2.85 bar, it is observed in Figure 7A that release time starts declining. We have to consider the integrated effect of *P*_atom_ and *T*_fluid_ to elaborate this finding. The escalated temperature causes the solvent in spray droplets to dry at a rapid pace since the size of spray droplets decreases with an increase in pressure. Consequently, a fraction of spray droplets undergoes pre-mature drying. Some of the dried particles are elutriated with fluidizing air and exit from the fluidized bed machine through its vent. This results in the formation of an irregular coating film. A portion of the quickly dried particles mixes with freshly introduced spay droplets and impinges on the surface of substrate particles. A collection of such pre-maturely dried particles makes the coating film porous and fluffy. Therefore, it is convenient for the nutrient molecules inside the granule core to diffuse through the grids of coating membrane due to enlarged voids. Hence, the release time decreases. A part of spray droplets goes through an increase in viscosity, due to the increased concentration of solids when fraction of solvent is rapidly evaporated. A sluggish droplet spreading on substrate surface due to higher viscosity results in the local accumulation of polymer material, which gives rise to coating heterogeneities. The higher the coating heterogeneities, the higher the release rate and decreased time for urea release. Figure 8 shows SCU samples that are achieved at (A) moderate (good quality SCU) and (B) extreme (poor quality SCU) conditions of temperature and pressure. The statistical analysis (ANOVA) and curvature of the contours in Figure 7A clearly indicate that *T*_fluid_ (*p*-value = 0.0001) is more influential and statistically significant parameters as compared to *P*_atom_ (*p*-value = 0.045).

The aforementioned results are in line with the results of similar studies (with different substrates) that are reported in literature. For example, Lan et al. reported that the formation of bigger spray droplets at lower atomizing pressure caused the urea substrate to dissolve, which led to a rough and porous coating film. High pressure also resulted in porous coating, while a mediocre pressure produced promising results with respect to permeability coefficient of nutrient [46]. Similarly, Rosa et al. reported that a significant growth of substrate particles was observed for atomizing pressure near the center points (moderate condition) [47]. Much lower and much higher temperatures were reported to have a negative impact on the growth of the coating film on the surface of urea granules when vinasse was used as a spray solution. A minimum release of nitrogen is reported for a temperature of 80 °C. The release time was found to decrease above 80 °C [20]. Similarly, the coating quality (and subsequently the release time) was found to have been improved with an increase in gas temperature up to 80 °C, followed by a deterioration of the film quality above 80 °C [48].

Figure 7B illustrates the integrated effect of *P*_atom_ and spray rate (*W*_s_) on the time of urea release from SCU. The spray rate is also a key parameter in the coating of urea granules in a fluidized bed. The spray rate determines the fluidized bed moisture since our coating solution is based on a waterborne biopolymer. In addition, the spray rate also determines the size of the droplets. It is believed that, for a given atomizing pressure, the mean size of the droplets increases with an increasing spray rate [27]. Better release properties are observed for a combination of *P*_atom_ and *W*_s_ at (or near) center points, that is, *P*_atom_ = 2.75 bar and *W*_s_ = 0.10 3L/s (2.75 RPM). A higher *P*_atom_ and *W*_s_ result in decreased time of urea release from SCU. *W*_s_ appears to be more influential (*p*-value = 0.002) as compared to *P*_atom_ (*p*-value = 0.040), as evident from statistical analysis and curvature of contours of response surface in Figure 7B. Therefore, the major role towards the reduced release time at the highest values of *P*_atom_ and *W*_s_ is attributed to the spray rate. At higher *W*_s_, spray droplets of relatively large size are produced, despite high pressure. For a given temperature, it takes longer for the solvent to evaporate from a bigger droplet as compared to a small one. Therefore, the delayed evaporation of solvent does not only give rise to preferential coating due to local accumulation, but it also forms rough and porous coating films when temporary agglomerates, developed between granules due to insufficient drying of spray droplets, are broken. Since urea is highly soluble in water, even the small pores on the membrane surface developed due to coating heterogeneities being enough to allow dissolved urea to have an easy escape through the grids of coating membrane, which leads to a reduced time for urea release. The same situation prevails at the lowest magnitudes of *P*_atom_ and *W*_s_, where the high droplet size is because of the lower *P*_atom_. These findings concur with Lan et al. [46], who reported: (a) very low spray rates result in poor spreading; (b) mediocre spray rates form a smoother dense coating; and, (c) higher spray rates form loose, porous coatings.

**Figure 9:**
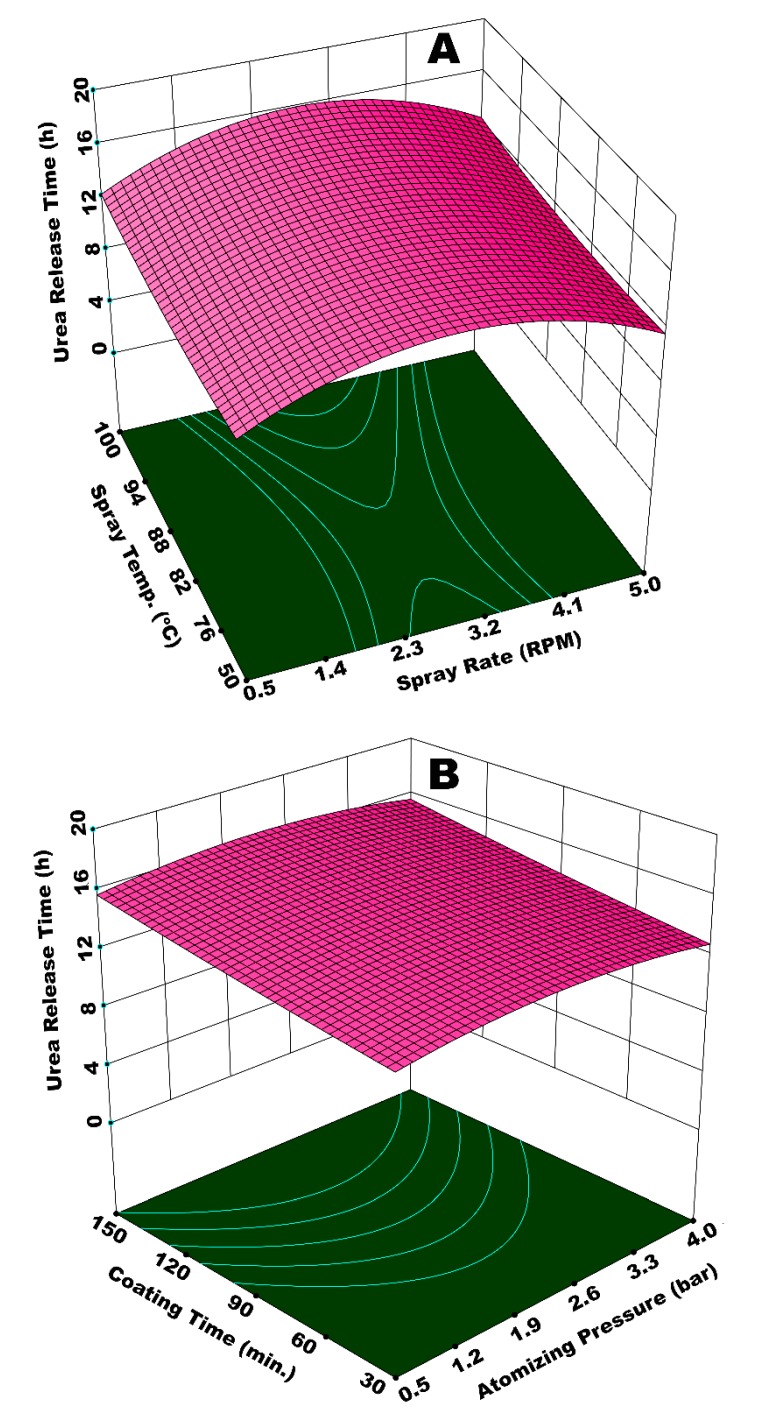
Response surface for (**A**) spray rate and spray temperature *vs* urea release time and (**B**) atomizing pressure and coating time *vs* urea release time.

Figure 9A illustrates the integrated effect of spray rate (*W*_s_) and spray temperature (*T*_spray_) on time of urea release from SCU. *W*_s_ determines the amount of moisture in fluidized bed, while *T*_spray_ contributes towards the overall dewatering capacity. The lowest value of release time is observed at the highest *W*_s_ and the lowest *T*_spray_. A diminished evaporation of solvent and increased viscosity of spray droplets at lower *T*_spray_ results in a lower rate of droplet impingement, poor droplet spreading, and, consequently enhanced coating heterogeneities on the surface of the substrate granules. A higher *W*_s_ on the other hand does not only reinforce the aforementioned discrepancies, but also results in spray loss on the walls. The integrated effect of higher *W*_s_ and lower *T*_spray_, therefore, results in porous and irregular membrane formation in addition to spray loss per unit time of the operation. Hence, urea molecules escape from irregular and wide membrane grids at a rapid pace during dissolution. A fraction of the substrate feed underwent temporary agglomeration due to the insufficient and delayed evaporation of solvent and adhesion properties of starch. The agglomerates disturbed the overall dynamics of the fluid bed by affecting the path and frequency of granules to come across the spray zone. The disturbance in fluid dynamics gave rise to coating heterogeneities. In addition, the temporary agglomerates, when broke, produced coated granules with rough and porous films. All of these factors are responsible for a lower release time of urea due to a poor coating uniformity and porous coating film. It is observed that the best results for urea release time are achieved at or near the centre points of experimental design, that is, *W*_s_ = 0.11 mL (2.75 RPM) and *T*_spray_ = 80 °C. These results are compatible with the literature results; for example, Naz et al. studied the effect of solution temperature on release time of urea coated with borax-modified starch and reported the longest release time of urea at 80 °C of spray solution [49]. The curvature of response surface contours in Figure 9A and statistical analysis (ANOVA) of the results indicate that *W*_s_ is more influential (*p*-value = 0.01) as compared to *T*_spray_ (*p*-value = 0.06), which is significant just close to the 95% confidence level.

Figure 9B illustrates the combined effect of *P*_atom_ and coating time (*t*_coat_) on the release time of urea from SCU. Although the combination of these two parameters is not exactly statistically significant to a confidence level of 95% (*p*-value = 0.062), it is important to discuss how significant a role the coating time plays. Normally, it is believed that an increase in coating time has a positive effect with respect to the growth of the substrate particles, giving rise to release time when coated particles are subjected to dissolution in water. However, this increase in particle growth and subsequent release time do not always have a linear relationship with coating time due to the interaction of other operating parameters.

It is observed in Figure 9B that the release time of urea increases with an increase in coating time, however the release time undergoes a decline afterwards. A fraction of the coating film starts disintegrating when substrate granules are subjected to coating session for extended time period due to hydrodynamics of the fluidized bed (continuous rolling motion of granules on rotary plate in this study). Even a minute size pore on the film surface is enough for the quick release of nutrient from SCU. Shortest release time of urea is witnessed at the lowest *P*_atom_ and *t*_coat_. With these conditions, it was observed that a very thin coating film was received on the surface of SCU granules. The surface of pristine urea was not fully covered, even for some granules. At minimum *P*_atom_ and *t*_coat_, the wetting, spreading, film formation, solvent evaporation, and development of final solid film do not go streamline. Relatively larger spray droplets at minimum *P*_atom_ experience poor spreading. For a given temperature, the granules find less time for the solvent to evaporate. Therefore, temporary agglomerates are developed that disturb the overall dynamics of the fluid bed. A short process time decreases the possibility of the granules to come across the spray zone. The integrated effect of minimum *P*_atom_ and *t*_coat_ does not only produce granules with thin and porous coating film, but some of the granules remain pristine, which results in lower release time.

**Figure 10:**
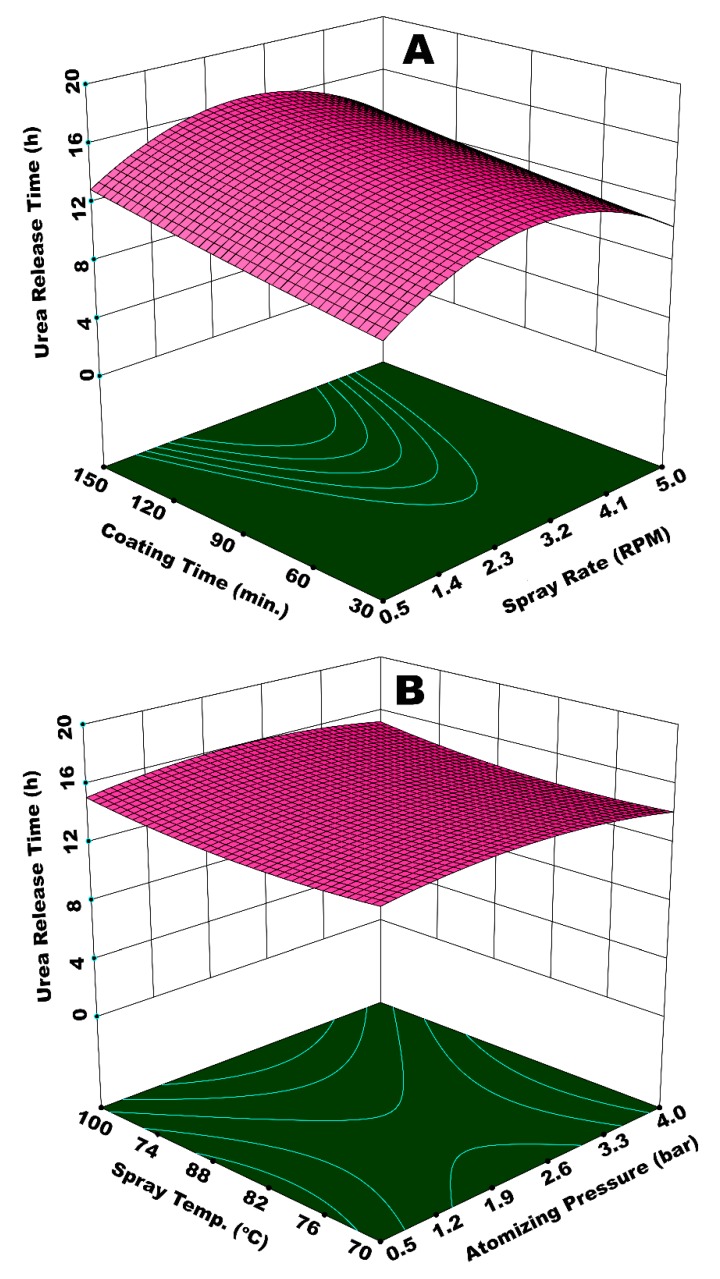
Response surface for (**A**) spray rate and coating time *vs* urea release time and (**B**) atomizing pressure and spray temperature *vs* urea release time.

Figure 10A shows a response surface illustrating the effect of *W*_s_ and *t*_coat_ on urea release time. The response surface resembles a hyperbolic shape, such that release time first increases with increase in both *t*_coat_ and *W*_s_, reaches to an apex at the foci of the hyperbolic paraboloid, and then declines after a further increase in *t*_coat_ and *W*_s_. Two positions on this response surface are indicative of the lowest release time; (i) at minimum values of *W*_s_ and *t*_coat_, and (ii) at maximum *W*_s_ and minimum *t*_coat_. At a maximum *W*_s_ and minimum *t*_coat_, the evaporation efficiency of the fluidizing air is reduced, and the relative humidity of fluidized-bed increases due to increased solvent input and lower dewatering capacity per unit time per unit area of the fluidized bed. Hence, the droplets are vulnerable to form agglomerates, which consequently increase the chances of preferential coating. In addition, the flow of the coating film on the particle surface is sluggish due to lower Reynolds number (lower velocity), which causes poor spreading [28]. Thus, part of the particle surface accumulates more coating material as compared to the rest of the surface, giving rise to coating heterogeneity. It is observed in our experiments that part of the particles with wet surfaces stick to the walls of fluidized bed for quite some time. This disturbs the hydrodynamics of the fluidized bed and results in preferential coating, which ultimately results in a reduced release time. In the case of minimum *W*_s_ and *t*_coat_, lower release time is because of the development of very thin membrane film and uncovered sites on surface of the urea granules mainly because of the shorter process time. The longest release time is observed for the highest *t*_coat_ (150 min) and a moderate *W*_s_ (0.11 mL/s). This is because of the growth of a significantly thick polymer film at a moderate pressure for longer coating time. There were hardly any losses on the walls of the fluidized bed in this case. The extended rolling motion of coated granules with thick coating film contributed towards the development of a robust film, which could resist the osmotic pressure of dissolved urea for a longer period of time. Hence, the release time increases in this case. These results are consistent with the literature results; for example, it is reported that coating mass and coating uniformity increase with coating time [50], which results in the longevity of nutrient release. However, it is observed in our study that a linear increase in coating mass and coating uniformity with increase in coating time strongly depends on the behavior of the interacting parameters. Figure 10B represents the integrated effect of *P*_atom_ and *T*_spray_ on urea release time. In this case, the curvature of response surface contours indicates only a slight impact on release time of urea by the interaction of *P*_atom_ and *T*_spray_. A dip in the response surface at minimum *P*_atom_ and *T*_spray_ indicates a lower release time, which can be characterized by the production of SCU granules with rough and porous coating film due to larger spray droplets and the poor evaporation efficiency of fluidizing air.

### 3.3. Interactive Effect of Process Parameters on Urea Release Kinetics

**Figure 11:**
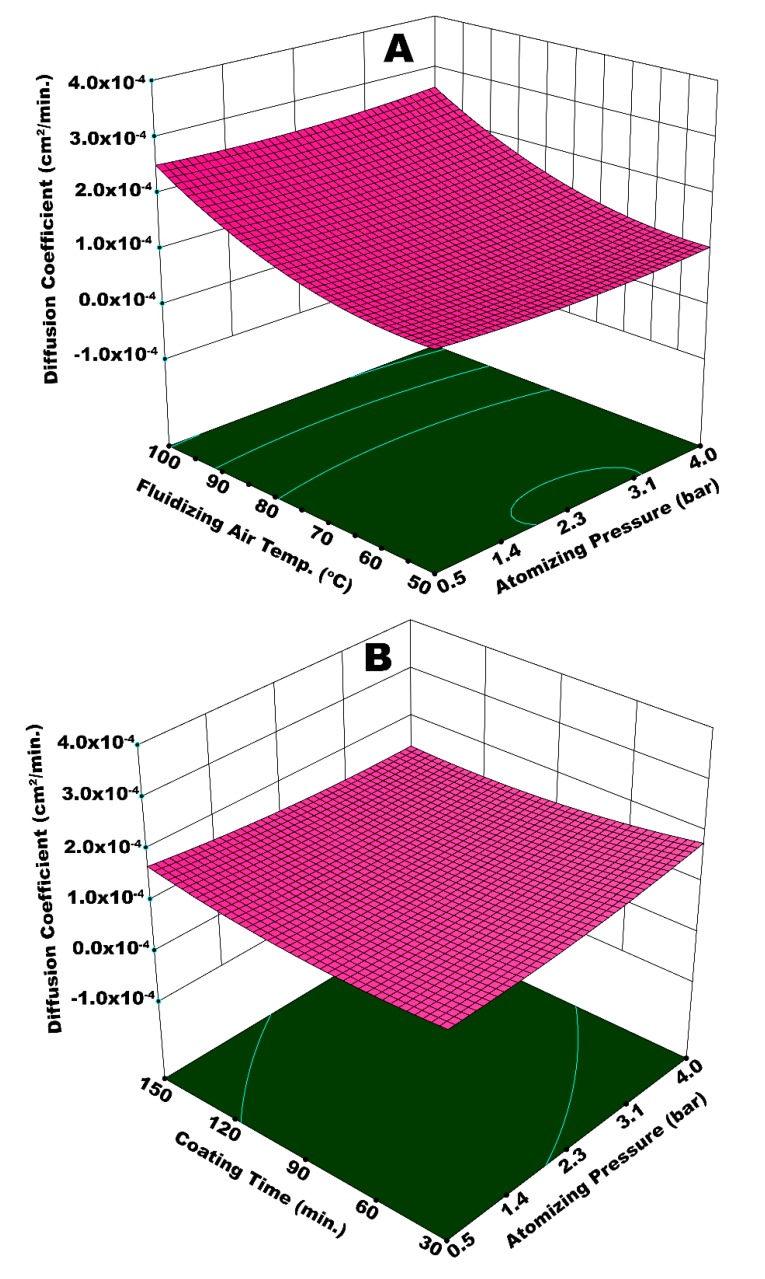
Response surface for (**A**) atomizing pressure and fluidizing air temperature *vs* diffusion coefficient and (**B**) atomizing pressure and coating time *vs* diffusion coefficient.

The release kinetics of urea while going through dissolution in water is reported in terms of the diffusion coefficient of nutrient. Figure 11A illustrates the interactive effect of *T*_fluid_ and *P*_atom_ on diffusion coefficient (*D*_c_). Principally, a good slow release urea is expected to have a lower *D*_c_, which refers to extended release time. The highest *D*_c_ is achieved at the maximum values of *T*_fluid_ and *P*_atom_, as evident from Figure 11A. Very fine spray droplets are generated at higher *P*_atom_, as explained earlier. A temperature as high as 100 °C causes quick and pre-mature drying of the fine droplets before their impingement on surface of the substrate. A fraction of dried particles escapes the fluid bed via elutriation and the rest of dried particles mingle with fresh spray droplets that succeed to impinge on the substrate surface. These particles resist the smooth spreading and film formation phenomena, which results in a porous, rough, and heterogeneous coating film. At such conditions of temperature and pressure, some of the granules were only partially engulfed by coating material, leaving parts of the surface uncovered. A poor coating film could hardly resist the release of urea molecules for long time. Hence, the diffusion coefficient was high in this case.

A higher *D*_c_ is also observed at a maximum *T*_fluid_ and a minimum *P*_atom_. It is difficult for the bigger spray droplets to spread on the surface of granules. Despite being bigger in size, the droplets are dried or partially dried before undergoing the spreading step giving rise to preferential coating since the temperature is high (100 °C). A non-uniform coating that results from preferential coating is responsible for an escalated *D*_c_. Significantly lower *D*_c_ is observed at a minimum *T*_fluid_ and maximum *P*_atom_. The solvent evaporation from very fine droplets generated at high pressure is relatively easy, despite a lower temperature. A thin, yet uniform, coating film achieved at these conditions resulted in a decrease in *D*_c_. The statistical analysis shows that *T*_fluid_ (*p*-value = 0.0087) was more influential when compared to *P*_atom_ (*p*-value = 0.049). Figure 11 (B) represents the diffusion coefficient as a function of *t*_coat_ and *P*_atom_ combined. The analysis of variance (ANOVA) indicates that the combination of *t*_coat_ and *P*_atom_ is not statistically significant (*p*-value = 0.065), with a confidence level of 95%. However, an important observation is made for *D*_c_ at the minimum *t*_coat_ and maximum *P*_atom_. The augmented value of *D*_c_ at these conditions is because of two situations: (i) the development of a very thin coating film at higher *P*_atom_ and (ii) the spray loss due to pre-mature drying and elutriation of fine droplets at a given fluidized bed temperature. The spray loss and subsequent poor film development led to increased *D*_c_.

**Figure 12:**
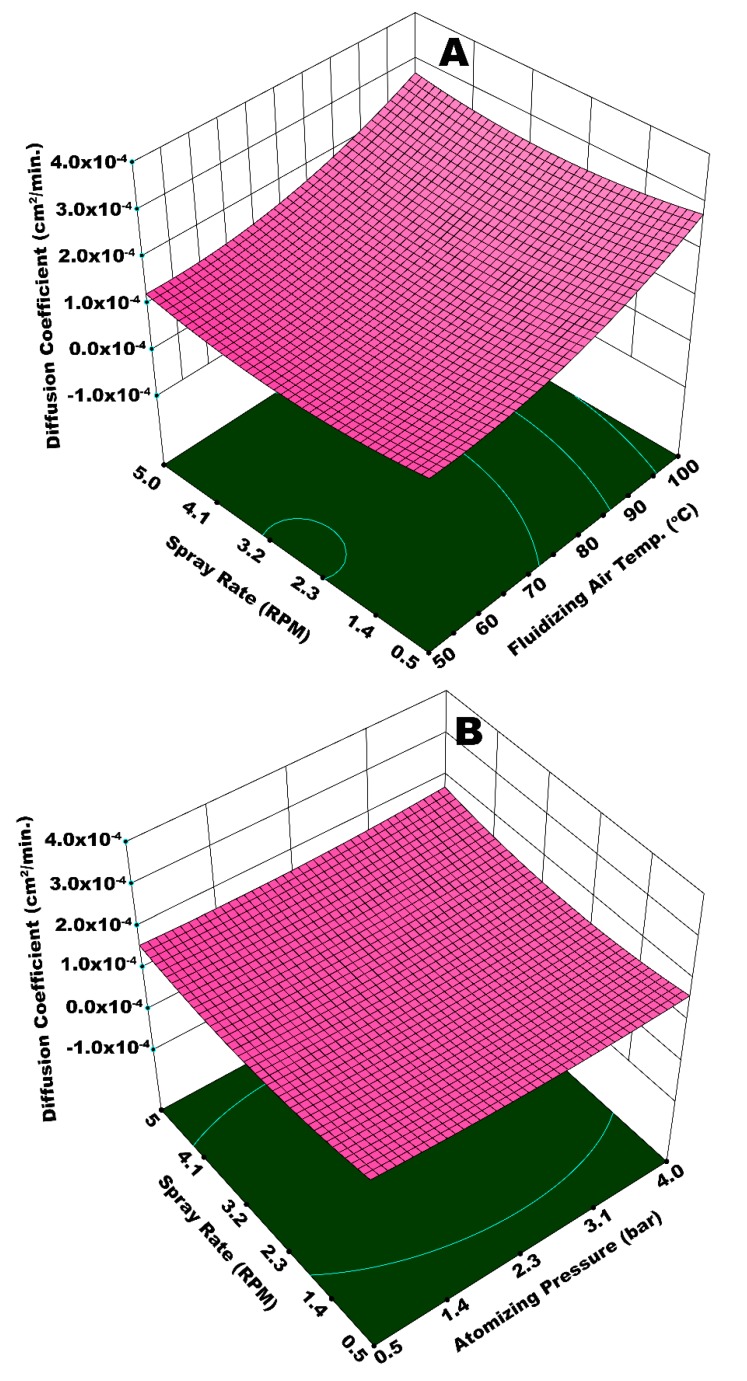
Response surface for (**A**) spray rate and fluidizing air temperature *vs* diffusion coefficient and (**B**) atomizing pressure and spray rate *vs* diffusion coefficient.

Figure 12A illustrates the integrated effect of *T*_fluid_ and *W*_s_ on the diffusion coefficient. Statistical analysis (ANOVA) suggests that *T*_fluid_ is more influential in this case (*p*-value = 0.0068) as compared to *W*_s_ (*p*-value = 0.019). A higher value of *D*_c_ is observed at the maximum magnitudes of *T*_fluid_ and *W*_s_. There can be two possible phenomena for this kind of a result. A higher spray input refers to an increased inflow of water content in the fluidized bed per unit time. This can potentially decrease the bed mobility, giving rise to preferential coating by limiting the frequency of substrate granules to come across the spray zone. It is reported that the spray rate also affects the droplet size, such that relatively large spray droplets are yielded at higher spray rate [27]. In the second possible situation, fluidizing air (at 100 °C) could have dried even the bigger droplets before they could undergo the proper spreading on the surface of granules. This local accumulation and consequent preferential coating results in coating heterogeneity and the development of irregular membrane film. In addition to the aforementioned situations, higher spray rate led to spray losses on the machine walls that disturbed hydrodynamics of the fluidized bed. All of these factors are responsible for a lowquality coating film and subsequent rise in *D*_c_. To the contrary, a significantly lower *D*_c_ was observed at the minimum values of *T*_fluid_ and *W*_s_. All the coating steps, that is, droplet impingement, coalescence, spreading, solvent evaporation, and solid film formation took place in a streamline manner, since *T*_fluid_ was more influential in this particular case and relatively fine droplets were produced at lower *W*_s_. A uniform coating film resulted at such conditions of temperature and spray rate. Hence, a lower *D*_c_ was resulted. A higher *D*_c_ for the maximum values of *P*_atom_ and *W*_s_ (Figure 12B) can be explained in terms of poor dewatering capacity of fluidizing air and enhanced spray losses at an escalated spray rate. It was expected that higher atomizing pressure would facilitate easy evaporation of fine droplets; however, spray rate is observed to have a dominant effect due to its overwhelming statistical significance (*p*-value = 0.009) as compared to *P*_atom_ (*p*-value = 0.055).

**Figure 13:**
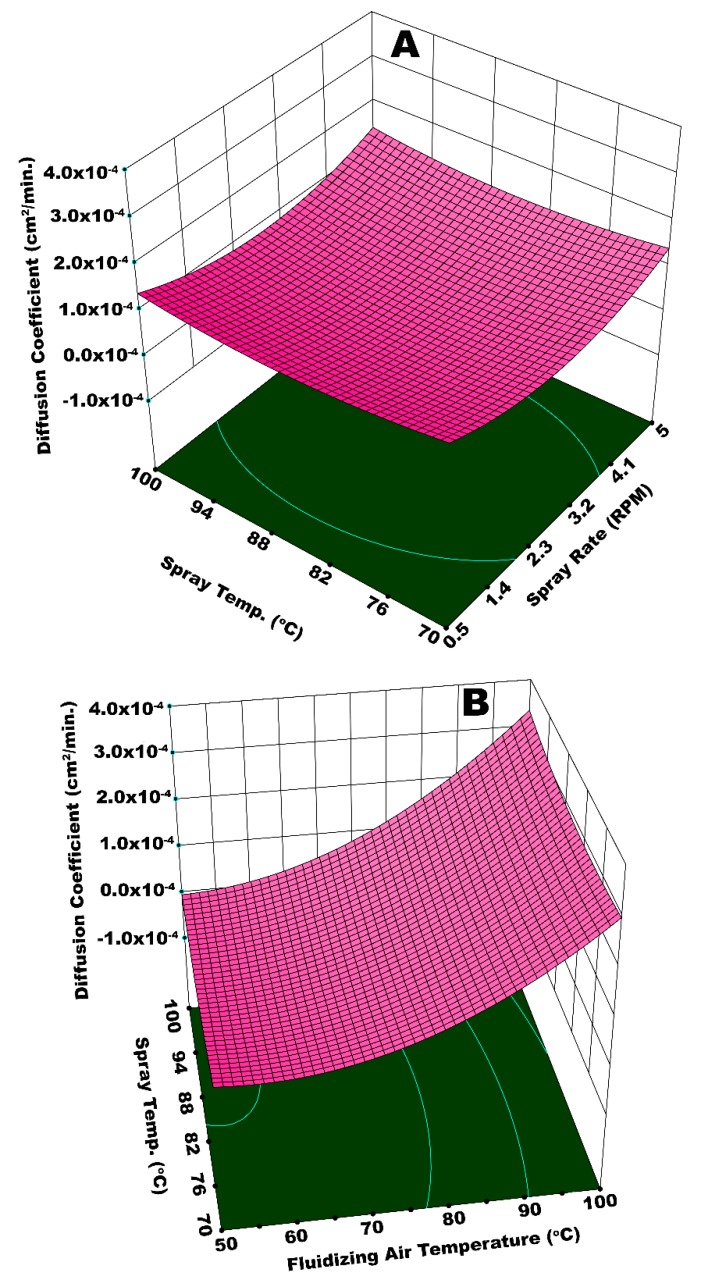
Response surface for (**A**) spray rate and spray temperature *vs* diffusion coefficient and (**B**) fluidizing air temperature and spray temperature *vs* diffusion coefficient.

Figure 13A illustrates the interactive effect of *W*_s_ and *T*_spray_ on the diffusion coefficient. In this case, the statistical analysis shows that *W*_s_ is more influential (*p*-value = 0.003) as compared to *T*_spray_ (*p*-value = 0.046). A minimum *D*_c_ is observed at *T*_spray_ = 70 °C and a minimum spray rate. At a given fluidizing air temperature, *T*_spray_ = 70 °C is sufficient for the appropriate and timely evaporation of solvent from relatively fine spray droplets generated at a lower spray rate. At these conditions, it is believed that all of the coating steps, namely, droplet impingement, droplet spreading, solvent evaporation, and solid film formation took place in a streamline manner to develop a fine and uniform coating film that withstood against the osmotic pressure of dissolved urea inside the core of urea granule for longer time during release experiment, resulting in a lower diffusion coefficient.

To the contrary, a higher *D*_c_ is achieved at the maximum values of *W*_s_ and *T*_spray_. A higher spray rate results in a higher moisture input into the system. In addition, it is believed that relatively bigger spray droplets are generated at an escalated spray rate. Two possible phenomena can be held responsible for a higher diffusion coefficient as a result of the interactive effect of *W*_s_ and *T*_spray_. Significant spray losses were observed at a higher spray rate. The adhesion of spray droplets on the walls of machine did not only cause spray losses, some of the granules were stuck with these droplets that disturbed the flow dynamics of the fluidized bed, giving rise to preferential coating. In addition, a 100 °C temperature of bigger spray droplets made them dry at a quicker pace before their proper spreading. This reinforced the phenomenon of preferential coating and consequently led to coating heterogeneities that ultimately resulted in a higher diffusion coefficient due to the poor resistance that was offered by the membrane grids during dissolution of urea molecules. Figure 13B presents an integrated effect of *T*_fluid_ and *T*_spray_. In this case, the highest *D*_c_ is observed at the maximum temperatures of spray and fluidizing air. At such high temperatures of *W*_s_ and *T*_spray_, the spray droplets are pre-maturely dried and elutriated with fluidizing air causing the spray losses. Some of these dried particles circulate in the fluidized bed, mingle with freshly introduced spray droplets, and impinge on the granules’ surface. These dried particles reduce the film spreading and result in the formation of a rough and porous film. The combined effect of spray loss, which leaves a portion of the granules uncovered, and rough and porous film on surface of granules, is responsible for the quick release of urea molecules giving rise to *D*_c_. However, a lower *D*_c_ is observed at a *T*_spray_ of 70 °C and a minimum *T*_fluid_. A fine coating film due to smooth impingement, spreading, film formation, solvent evaporation, and dried solid film barricades the escape of urea molecules from the membrane grids for a longer time. Hence, a lower diffusion coefficient is achieved.

The multi-objective optimization produced optimum values of fluidizing air temperature (80 °C), spray rate (0.13 mL/s), atomizing pressure (3.98 bar), process time (110 min.), and spray temperature (70 °C). These optimum process parameters can be used for the optimized production of controlled release coated urea in a rotary fluidized bed machine, particularly when a starchy material is utilized as a coating material. This set of optimum conditions may be helpful to lay a foundation for the up-scaled production.

### 3.4. Nitrogen Release Characteristics of Double Coated Urea

**Figure 14:**
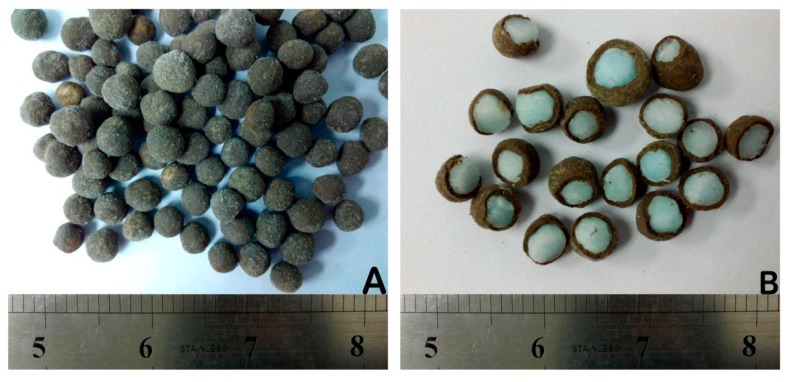
Double coated granules (**A**) and cross sections (**B**) of double coated urea (DCU).

**Figure 15:**
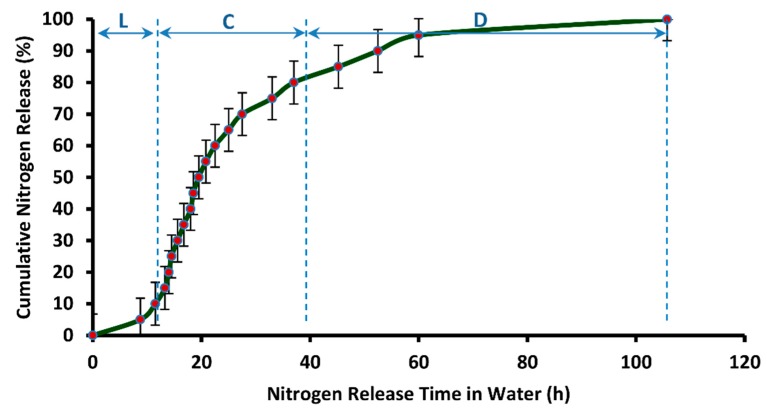
Cumulative release rate (in distilled water) vs. release time of nitrogen from DCU.

The SCU samples were subjected to double coating with external coating film of a geopolymer to produce double coated urea (DCU) in order to improve the release performance. After coating and drying steps, the DCU samples were subjected to water dissolution and soil burial tests for the evaluation of nitrogen release performance. Figure 14 represents DCU granules (A) and their cross sections (B). Figure 15 represents a plot of cumulative release rate versus release time of nitrogen resulted from water dissolution test of DCU granules. The mean of release time in water from five samples is reported.

It can be observed in Figure 15 that the release time of nitrogen from DCU (105.75 h or 4.4 days) is much higher than SCU, that is, single coated urea produced while using PVOH-modified starch material as coating solution. This is because of the augmented water barring efficiency of the dual coating layers. The release profile that is shown in Figure 15 follows the three stage release mechanism that was proposed by Liu et al. [51] and Shaviv et al. [52] for coated fertilizers, and it is also termed as the multi-stage diffusion model. According to this model, water penetrates the coating to condense on the solid fertilizer core, followed by partial nutrient dissolution. The driving force at this stage is the partial pressure of water vapor between the inside and outside of the coating film. This is termed as the lag-period and “L” in Figure 15 represents it. In this particular case, a lag period of 8.75 h is observed. Subsequently, as osmotic pressure builds within the containment, the membrane withstands the developing pressure, core fertilizer is thought to be slowly released via diffusion for which the driving force might be a concentration or pressure gradient, or combination thereof, called the “diffusion mechanism”. This is termed as constant-release-period. In Figure 15, “C” depicts the constant release period and this period is equal to 18.75 h. In third stage, also termed as the decay-period, release rate becomes very slow again, due to declined concentration gradient, and it is denoted by “D” in Figure 15. The decay period continues for 78.25 h (3.26 days).

**Figure 16:**
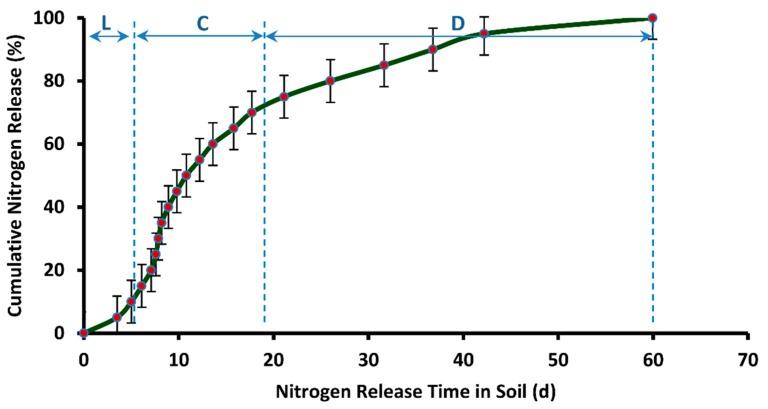
Cumulative release rate (in soil) vs. release time of nitrogen from DCU.

Figure 16 represents a plot of cumulative release rate versus release time of nitrogen resulted from the soil burial test of DCU granules. The mean of release time in soil from five samples is reported. The release profile indicates much better results with respect to release performance. The nitrogen release prolonged for as long as 60 days from the day of burial in the soil. Three distinct phases of nitrogen release are also observed in this case. In the first phase, represented as “L” in Figure 16, the nitrogen release is very slow. This phase is termed as Lag Period and it spans from the beginning until 3.5 days. In the second phase, an almost constant rate of nitrogen release is observed. This phase is termed as Constant Release Stage, and it spans from 3.5 days until 17.7 days. This phase is followed by Decay Period, in which the rate of nitrogen release declines to a much greater extent. This stage of nitrogen release in soil spans 17.7 days until 60 days. It is observed from Figure 16 that it takes 6.10 days for the release of first 15% of nitrogen from DCU in soil. According to European Standard (EN 13266, 2001), not more than 15% nutrient should release in 24 h from a controlled release fertilizer. Hence, the current result of nitrogen release from DCU is much better with respect to release performance. Table 3 presents a comparison of nitrogen release efficiency of DCU with the nitrogen release efficiency of double coated urea reported in the literature. The release performance of DCU is better in most cases, despite the use of synthetic polymers for double coating of urea, as reported in the literature.

### 3.5. Empirical Modelling of Nitrogen Release Kinetics

An empirical model is presented to correlate diffusion coefficient with time for 100% release of nitrogen from SCU. The investigation of diffusion coefficients and release kinetics is imperative in designing the new controlled release fertilizers and choosing the coating material of desired controlled release characteristics [57]. The phenomenon of nutrient transport involving molecular diffusion requires the knowledge about diffusion coefficient, which is often non-constant [23]. Most believe that the diffusion mechanism governs nutrient release from polymer-coated devices [58]. Hence, nutrient-release characteristics are generally characterized by determining the diffusion coefficient.

**Figure 17:**
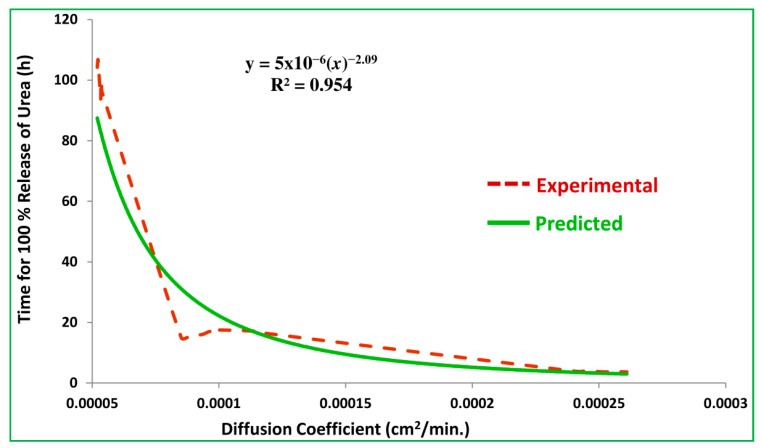
Plot of experimental and predicted values of *T*_100_*_._*

For the construction of this empirical model, the time for 100% release of nitrogen (*T*_100_) from the SCU samples is plotted against the corresponding diffusion coefficients, as shown in Figure 17. The power function curve fitting is accomplished, which produced the following empirical equation with a regression coefficient, R^2^ = 0.954.

(3) $$y=5\times 10^{-6}\left(x^{-2.098}\right)$$

where, *y* is the time for 100% release of nitrogen (min.) and *x* is the diffusion coefficient (cm^2^/min).

The empirical equation is used to predict the time for 100% release of nitrogen against the experimental values of diffusion coefficient. The experimental and predicted values of *T*_100_ are correlated and the coefficient of correlation equals 0.988, which represents how close the experimental and predicted data are. It also represents the accuracy of the proposed empirical model given in the empirical equation that can be used to estimate *T*_100_ for a known value of the diffusion coefficient. Similarly, it can also be used to estimate the diffusion coefficient for a given *T*_100_ of the starch based CRU.

## 4. Conclusions

The statistical analysis indicates that fluidizing air temperature and spray rate played the most influential role in describing the interactive effect of fluidized bed process parameters on the release characteristics of controlled-release urea that was produced in a rotary fluidized bed using modified-starch biopolymer as a coating solution. In any case, the longevity and kinetics of urea release were found to be functions of film thickness and intra- and inter-particle coating uniformity. The optimum process conditions can be used to design a rotary fluidized bed for the scaled-up production of CRU while using a viscous material like starch.

## Figures and Tables

**Table 1:** Characteristics of Fluidized Bed Machine.

Characteristics	Dimensions/Type
Internal Diameter	22 cm
Wall Thickness	0.7 cm
Nozzle Orifice Size (inner)	0.4 mm
Nozzle Orifice Size (outer)	0.7 mm
Spray Pattern	Full circular cone (Right-left spray)
Spray Angle	45 degree
Atomizer Type	Small particle mist generator
Fluid	Two-fluid mixed

**Table 2:** Acronyms, units, and maximum and minimum values of selected process variables.

Sr	Process Parameters	Acronym	Units	Min. Value	Max. Value
1	Atomizing air pressure	*P*_atom_	Bar	0.5	4.0
2	Fluidizing gas temp.	*T*_fluid_	°C	50	120
3	Spray rate	*W*_s_	mL/s	0.02	0.2
4	Spray temperature	*T*_spray_	°C	70	100
5	Coating time	*t*_coat_	Min	30	150

**Table 3:** Release performance of controlled release urea (CRU) coated with multiple materials.

Coating Material			Release Time in Water	Release Time in Soil	% Release	Ref
Inner Layer	Middle Layer	Outer Layer				
Modified Starch	-	Geopolymer	4.42 d	60 days	100%	Current Study
Polystyrene	-	Polyacrylic acid-containing urea	6 d	30 days	>90%	[17]
Polyurethane	-	Acrylic acid + chicken feather protein	2 d	56 days	~90%	[53]
Ethyl cellulose	-	Poly(acrylic acid–*co*–acrylamide)	-	30 days	100%	[54]
Wheatstraw + Sodium alginate	-	Poly(acrylic acid–*co*–*N* hydroxyl methyl acrylamide)	-	30 days	100%	[55]
k-carrageenan-sodium alginate	-	Acrylic acid + acrylamide	-	25 days	94%	[39]
Starch	-	Acrylic acid + acrylamide	-	30 days	61%	[56]
Urea formaldehyde	-	Polyacrylic acid/organoattapulgite	-	30 days	75%	[18]
Polyethylene	Poly(acrylic acid–*co*–acrylamide)	Poly(butyl methacrylate)	-	14 days	56%	[5]

## References

[1] Azeem B., Kushaari K., Man Z.B., Basit A., Thanh T.H. (2014). Review on materials & methods to produce controlled release coated urea fertilizer. J. Control. Release.

[2] Naz M.Y., Sulaiman S.A. (2016). Slow release coating remedy for nitrogen loss from conventional urea: A review. J. Control. Release.

[3] Salman O.A., Hovakeemian G., Khraishi N. (1989). Polyethylene-coated urea. 2. Urea release as affected by coating material, soil type and temperature. Ind. Eng. Chem. Res..

[4] Yang Y.C., Zhang M., Li Y., Fan X.H., Geng Y.Q. (2012). Improving the quality of polymer-coated urea with recycled plastic, proper additives, and large tablets. J. Agric. Food Chem..

[5] Tao S., Liu J., Jin K., Qiu X., Zhang Y., Ren X., Hu S. (2011). Preparation and characterization of triple polymer-coated controlled-release urea with water-retention property and enhanced durability. J. Appl. Polym. Sci..

[6] Azeem B., KuShaari K., Man Z., Trinh T.H. (2017). Nutrient release characteristics and coating homogeneity of biopolymer coated urea as a function of fluidized bed process variables. Can. J. Chem. Eng..

[7] Versino F., Urriza M., García M.A. (2020). Cassava-based biocomposites as fertilizer controlled-release systems for plant growth improvement. Ind. Crops Prod..

[8] Elhassani C.E., Essamlali Y., Aqlil M., Nzenguet A.M., Ganetri I., Zahouily M. (2019). Urea-impregnated HAP encapsulated by lignocellulosic biomass-extruded composites: A novel slow-release fertilizer. Environ. Technol. Innov..

[9] Pang L., Gao Z., Feng H., Wang S., Wang Q. (2019). Cellulose based materials for controlled release formulations of agrochemicals: A review of modifications and applications. J. Control. Release.

[10] Feng G., Ma Y., Zhang M., Jia P., Hu L., Liu C., Zhou Y. (2019). Polyurethane-coated urea using fully vegetable oil-based polyols: Design, nutrient release and degradation. Prog. Org. Coat..

[11] Azeem B., KuShaari K., Man Z., Irfan S.A. (2018). Parametric study of tumbling fluidized bed to evaluate nitrogen release characteristics of biopolymer-coated controlled release urea. Chem. Eng. Commun..

[12] Azeem B., KuShaari K., Man Z., Trinh T.H. (2018). Effect of fluidized-bed process variables on controlled-release of nitrogen and coating. Braz. J. Chem. Eng..

[13] Naz M.Y., Sulaiman S.A. (2014). Testing of starch-based carbohydrate polymer coatings for enhanced urea performance. J. Coat. Technol. Res..

[14] Tomaszewska M., Jarosiewicz A. (2004). Polysulfone coating with starch addition in CRF formulation. Desalination.

[15] Lum Y.-H., Shaaban A., Mohamad N., Dimin F., Yatim N.M. (2016). Boric acid modified starch polyvinyl alcohol matrix for slow release fertilizer. e-Polymers.

[16] Azeem B., KuShaari K., Man Z., Irfan S.A., Trinh T.H. (2017). Tumbling fluidized-bed process parameters affecting quality of biopolymer coating on surface of pristine urea particles. Powder Technol..

[17] Liang R., Liu M. (2006). Preparation and properties of a double-coated slow-release and water-retention urea fertilizer. J. Agric. Food Chem..

[18] Liang R., Liu M. (2006). Preparation and properties of coated nitrogen fertilizer with slow release and water retention. Ind. Eng. Chem. Res..

[19] Rajsharad C., Kamble S. (2006). Pva Based Film Coating and Film Coating Compositions. https://patents.google.com/patent/WO2006111980A2/en.

[20] Tsai B.S.E. (1986). Continuous Spouted Bed Process for Sulphur-Coating Urea. https://open.library.ubc.ca/cIRcle/collections/ubctheses/831/items/1.0058837.

[21] Basit A., KuShaari K., Trinh T.H., Azeem B. (2014). Spreading of Low Impact Velocity Droplet on Porous Surface. Int. J. Chem. Eng. Appl..

[22] Xie L., Liu M., Ni B., Zhang X., Wang Y. (2011). Slow-release nitrogen and boron fertilizer from a functional superabsorbent formulation based on wheat straw and attapulgite. Chem. Eng. J..

[23] Shavit U., Shaviv A., Zaslavsky D. (1995). Solute Diffusion-Coefficient in the Internal Medium of a New Gel Based Controlled-Release Fertilizer. J. Control. Release.

[24] Izhar S.M.M., Ku Shaari K.Z., Man Z., Samsudin Y.N. (2014). Influence of Citric Acid and Curing Time on Water Uptake. Appl. Mech. Mater..

[25] Hede P.D., Bach P., Jensen A.D. (2008). Two-fluid spray atomisation and pneumatic nozzles for fluid bed coating/agglomeration purposes: A review. Chem. Eng. Sci..

[26] Waldie B. (1991). Growth mechanism and the dependence of granule size on drop size in fluidized-bed granulation. Chem. Eng. Sci..

[27] Saleh K., Guigon P. (2006). Coating and Encapsulation Processes in Powder Technology. Granulation.

[28] Suzzi D., Radl S., Khinast J.G. (2010). Local analysis of the tablet coating process: Impact of operation conditions on film quality. Chem. Eng. Sci..

[29] Overbeek A. (2010). Polymer heterogeneity in waterborne coatings. J. Coat. Technol. Res..

[30] Dewettinck K., Messens W., Deroo L., Huyghebaert A. (1999). Agglomeration Tendency during Top-Spray Fluidized Bed Coating with Gelatin and Starch Hydrolysate. LWT Food Sci. Technol..

[31] Christoph Link K., Schlünder E.-U. (1997). Fluidized bed spray granulation: Investigation of the coating process on a single sphere. Chem. Eng. Process. Process Intensif..

[32] Keningley S.T., Knight P.C., Marson A.D. (1997). An investigation into the effects of binder viscosity on agglomeration behaviour. Powder Technol..

[33] Schaafsma S.H., Vonk P., Segers P., Kossen N.W.F. (1998). Description of agglomerate growth. Powder Technol..

[34] Rychter P., Kot M., Bajer K., Rogacz D., Šišková A., Kapuśniak J. (2016). Utilization of starch films plasticized with urea as fertilizer for improvement of plant growth. Carbohydr. Polym..

[35] Xiong H., Tang S., Tang H., Zou P. (2008). The structure and properties of a starch-based biodegradable film. Carbohydr. Polym..

[36] Yin Y., Li J., Liu Y., Li Z. (2005). Starch crosslinked with poly(vinyl alcohol) by boric acid. J. Appl. Polym. Sci..

[37] Shi R., Bi J., Zhang Z., Zhu A., Chen D., Zhou X., Zhang L., Tian W. (2008). The effect of citric acid on the structural properties and cytotoxicity of the polyvinyl alcohol/starch films when molding at high temperature. Carbohydr. Polym..

[38] Ariyanti S., Man Z., Azmi B.M. (2012). Improvement of Hydrophobicity of Urea Modified Tapioca Starch Film with Lignin for Slow Release Fertilizer. Adv. Mater. Res..

[39] Wang Y., Liu M., Ni B., Xie L. (2012). κ-Carrageenan–Sodium Alginate Beads and Superabsorbent Coated Nitrogen Fertilizer with Slow-Release, Water-Retention, and Anticompaction Properties. Ind. Eng. Chem. Res..

[40] Sreedhar B., Sairam M., Chattopadhyay D.K., Rathnam P.A.S., Rao D.V.M. (2005). Thermal, mechanical, and surface characterization of starch-poly(vinyl alcohol) blends and borax-crosslinked films. J. Appl. Polym. Sci..

[41] Reddy N., Yang Y. (2010). Citric acid cross-linking of starch films. Food Chem..

[42] Menzel C., Olsson E., Plivelic T.S., Andersson R., Johansson C., Kuktaite R., Järnström L., Koch K. (2013). Molecular structure of citric acid cross-linked starch films. Carbohydr. Polym..

[43] Xiao C., Yang M. (2006). Controlled preparation of physical cross-linked starch-g-PVA hydrogel. Carbohydr. Polym..

[44] Das K., Ray D., Bandyopadhyay N.R., Gupta A., Sengupta S., Sahoo S., Mohanty A., Misra M. (2010). Preparation and characterization of cross-linked starch/poly (vinyl alcohol) green films with low moisture absorption. Ind. Eng. Chem. Res..

[45] Shi R., Zhang Z., Liu Q., Han Y., Zhang L., Chen D., Tian W. (2007). Characterization of citric acid/glycerol co-plasticized thermoplastic starch prepared by melt blending. Carbohydr. Polym..

[46] Lan R., Liu Y., Wang G., Wang T., Kan C., Jin Y. (2011). Experimental modeling of polymer latex spray coating for producing controlled-release urea. Particuology.

[47] Da Rosa G.S., Dos Santos Rocha S.C. (2013). Use of vinasse to produce slow-release coated urea in spouted bed. Can. J. Chem. Eng..

[48] Weiss P.J., Meisen A. (1983). Laboratory Studies on Sulfur-Coating Urea by the Spouted Bed Process. Can. J. Chem. Eng..

[49] Naz M.Y., Sulaiman S.A., Ariwahjoedi B., Shaari K.Z.K. (2015). Effect of pre-coat solution temperature on fluidized bed urea coatings. Surf. Eng..

[50] Abe E., Yamada N., Hirosue H., Nakamura H. (1998). Coating mass distributions of seed particles in a tumbling fluidized bed coater. Powder Technol..

[51] Liu L., Kost J., Fishman Marshall L., Hicks Kevin B. (2008). A Review: Controlled Release Systems for Agricultural and Food Applications. https://pubs.acs.org/doi/abs/10.1021/bk-2008-0992.ch014.

[52] Shaviv A. (2005). Controlled release fertilizers. Int. Work. Enhanc. Fertil..

[53] Yang Y.C., Tong Z.H., Geng Y.Q., Li Y.C., Zhang M. (2013). Biobased Polymer Composites Derived from Corn Stover and Feather Meals as Double-Coating Materials for Controlled-Release and Water-Retention Urea Fertilizers. J. Agric. Food Chem..

[54] Ni B., Liu M., Lü S., Xie L., Wang Y. (2011). Environmentally friendly slow-release nitrogen fertilizer. J. Agric. Food Chem..

[55] Xie L., Liu M., Ni B., Wang Y. (2012). New environment-friendly use of wheat straw in slow-release fertilizer formulations with the function of superabsorbent. Ind. Eng. Chem. Res..

[56] Guo M., Liu M., Zhan F., Wu L. (2005). Preparation and properties of a slow-release membrane-encapsulated urea fertilizer with superabsorbent and moisture preservation. Ind. Eng. Chem. Res..

[57] Paulo Filho M., Rocha S.C.S., Lisboa A.C.L. (2006). Modeling and experimental analysis of polydispersed particles coating in spouted bed. Chem. Eng. Process. Process Intensif..

[58] Peppas N.A. (1985). Analysis of Fickian and non-Fickian drug release from polymers. Pharm. Acta Helv..

